# Systematic Review of Pre-injury Migraines as a Vulnerability Factor for Worse Outcome Following Sport-Related Concussion

**DOI:** 10.3389/fneur.2022.915357

**Published:** 2022-06-20

**Authors:** Douglas P. Terry, Fionn Büttner, Nathan A. Huebschmann, Andrew J. Gardner, Nathan E. Cook, Grant L. Iverson

**Affiliations:** ^1^Vanderbilt Sports Concussion Center, Department of Neurological Surgery, Vanderbilt University Medical Center, Nashville, TN, United States; ^2^Department of Medical Statistics, London School of Hygiene and Tropical Medicine, London, United Kingdom; ^3^School of Public Health, Physiotherapy and Sports Science, University College Dublin, Dublin, Ireland; ^4^Grossman School of Medicine, New York University, New York, NY, United States; ^5^Priority Research Center for Stroke and Brain Injury, School of Medicine and Public Health, University of Newcastle, Callaghan, NSW, Australia; ^6^Department of Physical Medicine and Rehabilitation, Harvard Medical School, Boston, MA, United States; ^7^Department of Physical Medicine and Rehabilitation, Spaulding Rehabilitation Hospital, Charlestown, MA, United States; ^8^MassGeneral Hospital for Children Sports Concussion Program, Boston, MA, United States; ^9^Department of Physical Medicine and Rehabilitation, Spaulding Research Institute, Charlestown, MA, United States

**Keywords:** migraine, concussion, sport-related concussion, traumatic brain injury, systematic review, prolonged recovery

## Abstract

**Background:**

Individuals with migraine disorders may be affected differently by concussions compared to individuals without migraine disorders. Prior studies on this topic have had mixed results. The purpose of this study was to systematically examine clinical outcomes following a sport-related concussion in athletes who have a pre-injury history of migraines.

**Methods:**

All studies published prior to 15 May 2021 that examined pre-injury migraines as a possible predictor of clinical recovery from concussion were included. The search included (i) sport/athlete-related terms, (ii) concussion-related terms, and (iii) diverse predictor/modifier terms. After removing duplicates, 5,118 abstracts were screened, 538 full-text articles were reviewed, and 27 articles were included for narrative synthesis without meta-analysis (*n* = 25 with unique samples). Risk of bias was assessed using the domain-based Quality In Prognosis Studies (QUIPS) tool.

**Results:**

Most studies did not find pre-injury migraines to be associated with concussion outcome, but several of these studies had small or very small sample sizes, as well as other methodological weaknesses. Risk of bias varied greatly across studies. Some of the larger, better-designed studies suggested pre-injury migraines may be a risk factor for worse concussion outcome. Most articles examined pre-injury migraines as an exploratory/secondary predictor of concussion outcome; very few were designed to examine migraine as the primary focus of the study. Migraine history was predominantly based on self-report and studies included minimal information about migraine (e.g., age of onset, frequency/severity, past treatment). Effect sizes were usually not reported or able to be calculated from reported study data.

**Conclusion:**

There is some evidence to suggest that pre-injury migraines may be a vulnerability factor for a worse outcome following concussion, with studies having the lowest risk of bias reporting a positive association. Future studies should focus on improving methodological quality when assessing the relationship between pre-injury migraines and concussion outcome and better characterizing pre-injury migraine status.

**Systematic Review Registration:**

https://www.crd.york.ac.uk/prospero/display_record.php?ID=CRD42019128292, identifier: PROSPERO 2019 CRD42019128292.

## Introduction

There is considerable interest in better understanding factors that may influence recovery following sport-related concussion, including pre-existing health conditions, injury-related factors, and acute symptomatology (1–3). A pre-injury history of migraine headaches is one such factor. Migraine headaches, which are commonly characterized by a unilateral throbbing sensation accompanied with light/noise sensitivity, nausea, and vomiting (4) occur in approximately 8% of youth (5) and 14% of adults (6). Middle school student athletes with migraines have a higher prevalence of prior concussion compared to those without migraines (31% vs. 13%) (7), suggesting that youth with migraines may be at a greater risk for sustaining concussion. This finding is important because individuals who have sustained prior concussions are at a higher risk of sustaining a future concussion (8) and having multiple concussions is associated with greater baseline symptom reporting (9) and a subset may be at increased risk for experiencing persistent symptoms following a subsequent concussion (10). Furthermore, athletes with pre-existing migraine disorders, regardless of prior concussion history, report more symptoms at baseline in the absence of a recent concussion (9). They also are more likely to endorse symptom provocation on vestibular/ocular-motor screening tests, in the absence of a concussion, during preseason baseline testing (11). Moreover, they report more symptoms acutely following a concussion than athletes without migraine histories (12). These findings indicate that individuals with migraine disorders may be affected differently by concussions compared to individuals without migraine disorders.

A 2017 systematic review examining a variety of factors that may be associated with clinical outcome following a concussion (1) identified 10 studies published prior to June 2016 that analyzed whether having pre-injury migraines was a risk factor for protracted recovery following concussion (10, 13–21). Pre-existing migraine history was not a primary risk factor of interest in nearly all of these studies. Only one study (10) showed that migraine history is associated with worse clinical outcome; a greater proportion of youth with pre-injury migraines (43%) experienced persistent post-concussive symptoms at 28 days post-injury compared to those without pre-injury migraines (28%). However, significant variability in the methodology of these studies may have influenced the conclusions of the prior systematic review (1). Moreover, the prior systematic review focused broadly on any and all potential risk factors for worse outcome and did not examine any individual risk factor in depth. Additionally, many studies examining risk factors for a worse clinical recovery have been published since 2017, necessitating an updated review. Thus, the purpose of the present systematic review is to provide a thorough and targeted examination of whether pre-injury migraines may be a vulnerability factor for a protracted recovery following concussion, characterize potential gaps in the literature, and propose ways to advance our understanding of the association between pre-injury migraine and clinical outcome following sport-related concussion. In addition to the aforementioned goals, this systematic review expands on the prior review because it includes additional studies published since June 2016 and evaluates the risk of bias for the included studies.

## Materials and Methods

The original systematic review (1) protocol was prospectively registered in the PROSPERO database for systematic reviews (protocol ID: CRD42016041479). The updated systematic review protocol was also registered (protocol ID: CRD42019128292). This systematic review is consistent with the guidelines from the Preferred Reporting Items for Systematic Reviews and Meta-Analyses (PRISMA) Statement (22).

### Search Strategy

**Figure 1:**
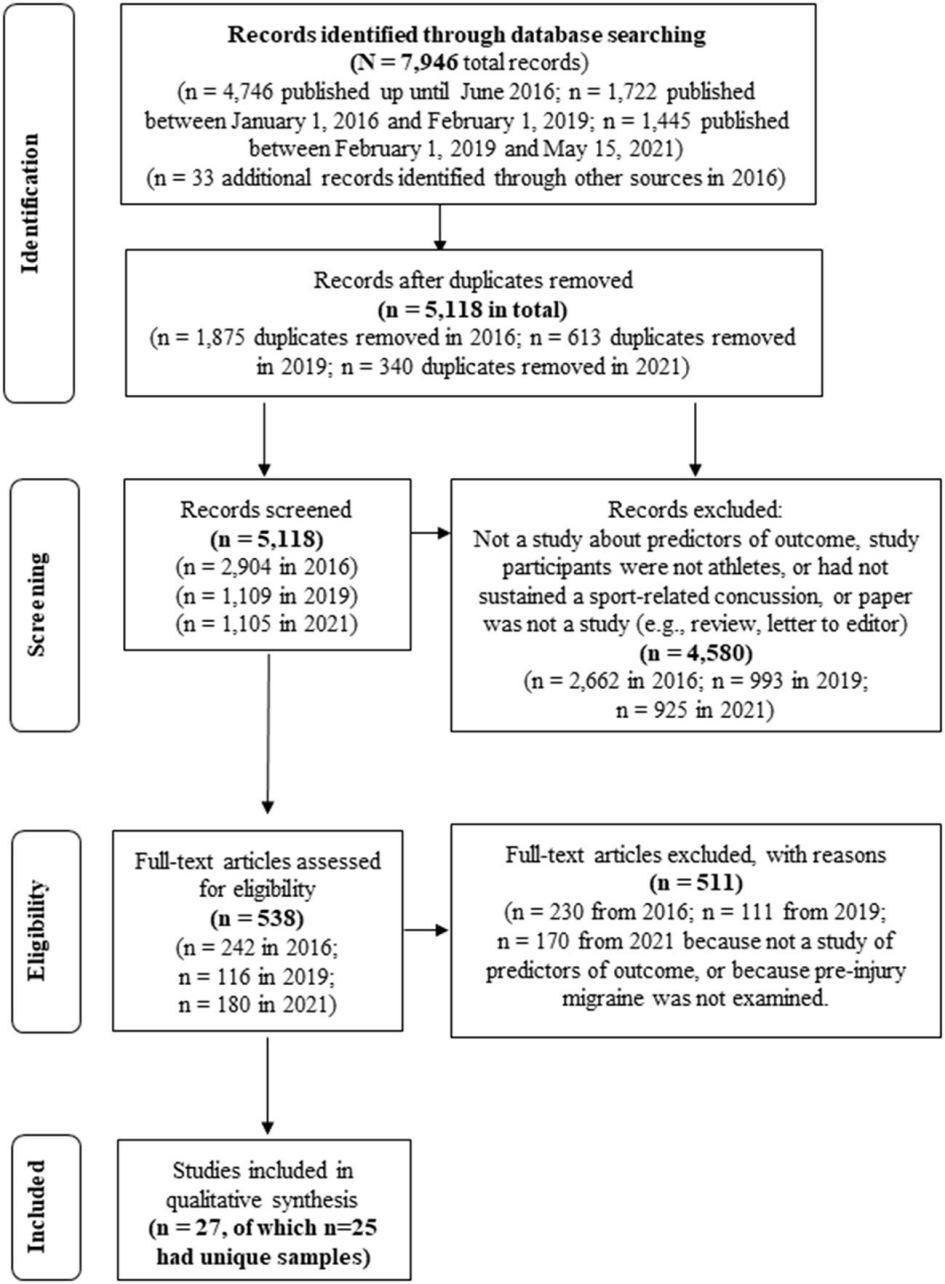
Systematic Literature Search (PRISMA).

Articles were identified by online database searching, by hand-searching reference lists of identified studies, and by performing cited reference searches (see Figure 1). The online databases that were searched included PubMed, MEDLINE®, PsycINFO®, CINAHL, Cochrane Library, EMBASE, SPORTDiscus, Scopus, and Web of Science. A total of three separate searches were conducted, each covering English-language articles published from (i) database inception to 1 June 2016; (ii) 1 January 2016 to 1 February 2019; and (iii) 1 February 2019 to 15 May 2021. The searches were otherwise identical. The key search terms consisted of two main themes, (i) sport and athlete-related terms and (ii) brain concussion related terms, as follows: sport, sports [MeSH]), athletic, athlete, player and craniocerebral trauma, brain injuries, brain concussion, sports concussion, athletic injuries, mild traumatic brain injury, mTBI, traumatic brain injury, TBI, brain concussion, concussion, multiple concussions, repeated concussion, repetitive concussion, cumulative concussions, concussion history, brain damage, prognosis, outcome, recovery, risk factor, injury incidence, sex differences, gender, genetics, ApoE, BDNF, S100B, GFAP, severity, loss of consciousness, LOC, post-traumatic amnesia, PTA, amnesia, retrograde amnesia, seizure, seizures, learning disorder, ADHD, level of education, migraine, mental health, sleep disorders, medications, cervical injury, vestibular injury, psychological reactions, anxiety, depression, headaches, intractable headaches, magnetic resonance imaging, MRI, computed tomography, and CT. This search strategy was deliberately broad in order to replicate the methods employed in the original systematic review (1), and to ensure that relevant articles that did not explicitly mention “migraine” in the title/abstract were not accidentally excluded. In the original systematic review, we found that several articles examined multiple predictors, many of which were not discussed in the abstract and were, instead, discussed elsewhere in the manuscript, such as in the Results section or in tables or figures.

### Article Selection and Data Extraction

When the search was repeated for the purpose of this systematic review (1), we discovered an error such that 7,617 records were reported when there were in fact 4,746 records in the original search, as reflected in Figure 1 of this paper. For the original and third searches, each title and abstract were examined for inclusion by two authors (GI and AG). For the second search, titles and abstracts were reviewed by one author (GI). Articles were identified for possible inclusion if they (i) assessed humans, and (ii) examined if pre-injury migraine was associated with outcome following sport-related concussion (in any capacity—that is, as a primary prognostic, confounding, mediating, or effect modifying variable). For all studies identified during the initial title/abstract screening, the full-text article was downloaded and reviewed by two authors to determine whether it met criteria for inclusion (original search: AG and GI; second search: DT and AG; third search: DT and NC). Reference lists of previously published reviews on predictors of concussion recovery were also examined for articles that may fit the inclusion criteria.

Outcome from sport-related concussion was defined in a variety of ways based on the methodology of the studies included in the review (e.g., days until symptom-resolution/return to baseline, days to return to school or sports, cognitive recovery/return to baseline). Studies that examined acute symptomatology/outcome in the first several days following a concussion but did not address clinical recovery or persistent symptoms were excluded. Four authors (NC, AG, DT, and NH) completed the extraction of information from the articles (i.e., first author, year of publication, the PubMed identification number, number of total participants, number of concussed participants, proportion of female participants, age, setting, study design, definition of outcome, the period for assessing the clinical outcome, findings regarding the association between migraine and outcome, number of participants with pre-injury migraines, method/definition for assessing pre-injury migraines, number of migraines in the past year before the index concussion, age of onset of migraines, whether migraines were differentiated from other headaches types, findings/effect size, and family history of migraine).

### Risk of Bias and Level of Evidence

Risk of bias was rated independently for each article by two authors (DT and FB) using the Quality In Prognosis Studies (QUIPS) tool (23). QUIPS is a domain-based tool that evaluates risk of bias in prognostic studies, which can influence the direction and magnitude of study results. The authors developed a rating rubric before evaluating the studies. Discrepancies were resolved through discussion. The level of evidence for each article was rated independently by two authors (DT and AG) using the Oxford Classification for Evidence-Based Medicine (24).

### Qualitative Synthesis and Likelihood Ratio Estimation

Heterogeneous subpopulations, comparators, outcomes, recovery timeframes, and operational definitions of pre-injury migraine used across studies precluded quantitative aggregation in the form of meta-analysis. Therefore, we performed a synthesis without meta-analysis by descriptively synthesizing included studies and systematically reporting study findings by study design, participant subpopulation (e.g., age range), pre-injury migraine definition, recovery timeframe, and clinical outcome (25, 26).

In addition, we used a likelihood heuristic to assess the probability of observing a specific number of statistically significant and non-significant studies reporting an association between migraine history and clinical outcome. This heuristic yields a likelihood ratio (LR) that estimates the weight of the evidence in support of the null hypothesis (i.e., migraine history *is not* associated with clinical outcome following concussion) vs. the alternative hypothesis (i.e., migraine history *is* associated with worse clinical outcome) (27). The likelihood heuristic has been used to examine associations between sport-related concussion and clinical outcomes in prior systematic reviews (3, 28). To calculate LRs, an *a priori* alpha (e.g., 0.05) and statistical power value (e.g., 0.80) are selected. Next, the number of studies (*k*) that reported a statistically significant result for various outcomes (e.g., symptom duration or time to return to sports) are summed and entered as a proportion of the overall number of studies reporting on a specific outcome (*n*). We used an LR calculator (https://lakens.shinyapps.io/likelihood) that has been developed to assist in synthesizing results from multiple studies (27). Two sets of analyses, with varying parameters, were conducted. First, an alpha level of 0.05 and power level of 0.80 were selected, reflecting near optimal study designs. Second, an alpha level of 0.25 and power level of 0.45 were selected, which is more likely to capture shortcomings inherent to several of the study designs included in this systematic review. The LR effect size estimates were summarized as follows: LRs > 8 and 32 are considered benchmarks of moderate and strong evidence, respectively (27, 29).

## Results

As shown in Figure 1, the search revealed 7,946 articles. There were 5,118 unique articles after removing duplicates. Based on a review of the titles and abstracts of these articles, 4,580 were excluded because they did not examine sport-related concussion or were not an empirical research study [e.g., review articles, editorials, chapters, commentaries, letters to the editor). This narrowed the pool of articles to 538, which were downloaded and reviewed in full. A total of 27 articles met inclusion criteria. Detailed information about these studies is included in Tables 1, 2. Three of these articles were from the same research group and included an identical sample with similar analyses and findings (15, 30, 31). For summary purposes, these three studies were grouped and discussed as one study. For the 25 studies with unique samples, there were 8,692 athletes who sustained a sport-related concussion. At least 1,015 of these athletes reported a pre-injury history of migraines (11.8%; *n* = 1,015/8,582); one study did not report the number of individuals with pre-injury migraines (32)]. There was substantial methodological variability across studies (see Tables 1, 2). Study designs included prospective cohort studies (12/25; 48%), retrospective cohort studies (10/25, 44%), case-control studies (2/25; 8%), and a case series (1/25; 4%). Outcomes also varied. Many studies defined recovery based on symptom resolution (11/25; 44%; including studies that deemed persistent symptoms as “post-concussion syndrome”), while other studies defined outcome as return to play or school (7/25; 28%), clinical recovery (e.g., resolution of symptoms and cognitive and/or balance difficulties, 3/25; 12%), presence of a new psychiatric diagnosis (1/25; 4%), vestibular symptom changes (1/25; 4%), and self-reported perceived recovery time (2/25; 8%). Some studies reported two or more outcomes. Statistically, some studies examined recovery time as a continuous variable, while other studies created a dichotomous variable based on recovery at various timepoints (i.e., 10 days, 14 days, 21 days, 28 days, 60 days, 3 months).

**Table 1:** Study characteristics.

**First Author (Year) PMID**	**Total *N*, # concussed, % female; age (in years) (mean, SD, range); setting**	**Study design, outcome variable, findings, and effect size**	**Significant findings?**	**Time period**
Aggarwal (2017) 29058480	*N* = 118, 118, 29% Age: M = 15.75, SD = 1.17, Range = 13–19 Setting: university-based concussion clinic	Retrospective observational cohort study Outcome: time to return to play Findings: those with personal/family history of migraines did not have a longer concussion resolution time in a multiple variable cox proportional hazards regression, *p* = 0.62, HR = 0.19	No	Until recovery M = 28 days; Md = 17 days
Beauchamp (2019) 31050335	*N* = 311, 311, 35% Age: Md = 11.9, IQR = 9.1–14.2, Range = 6–18 Setting: emergency department	Longitudinal, prospective cohort study Outcome: being well (i.e., no concussion symptoms, no cognitive inefficiency, and optimal quality of life score) at 4 weeks and 12 weeks following injury. Findings: history of migraine was one of 19 candidate variables entered into a preliminary logistic regression, but was removed from subsequent analyses in a backward stepwise elimination of candidate variables because *p* > 0.25. Effect size = NR	No	4 weeks / 12 weeks
Eagle (2020) 32330895	*N* = 218, 218, 42.2% Age: M = 14.7, SD = 0.1 (early presentation); M = 14.9, SD = 0.2 late presentation); Range: 12–17 Setting: concussion clinic	Retrospective cohort study (chart review); (80% with sport-related concussions) Outcome: prolonged clinical recovery > 30 days based on symptom presentation at rest, cognitive/ocular/vestibular performance, and symptom onset following exertion Findings: patients with a history of headache or migraine (37.6% of the sample) had a higher likelihood of having a prolonged recovery in a multiple variable backward stepwise logistic regression (OR = 4.02, 95% CI = 1.49–10.87)	Yes	30 days (recovery variable)
Eisenberg (2013) 23753087	*N* = 235, 235, 43% Age: M = 14.3, SD = NR, Range = 11–22 Setting: emergency department	Prospective cohort study (64% with sport-related concussions) Outcome: time to symptom resolution Findings: those with migraines did not have prolonged symptoms, *p* = 0.74; recovery time for migraine disorders: Md = 13 days; all participants: Md = 13 days. Percent symptomatic at 7, 28, and 90 days: migraine group = 83, 35, 20%; all participants = 77, 32, 15%. Effect size = NR	No	90 days
Ellis (2015) 26359916	*N* = 174, 174, 38% Age: M = 14.2, SD = 2.34, Range = ≤ 19 years Setting: pediatric concussion program	Retrospective cohort study Outcome: presence of psychiatric sequelae (i.e., worsening of symptoms of a pre-existing psychiatric disorder, new suicide ideation, or presence of a new psychiatric disorder) vs. no psychiatric disorder Findings: those with migraines did not have a higher likelihood of psychiatric outcome (10% vs. 9%; *p* = 0.895). Effect size = NR	No	1 month+
Fehr (2019) 29084034	*N* = 549, 431 in analyses, 45.5% Age: M = 14.3, SD = 2.1, Range:10–18 Setting: sports medicine concussion clinic	Retrospective cohort study (chart review) Outcome: time to reach self-reported symptom recovery Findings: those with migraines did not have a prolonged recovery. Effect Size HR = 1.08 (0.71–1.63), *p* = 0.64	No	Md = 40 days (M = 62, SD = 64)
Hiploylee (2017) 27784191	*N* = 285, 110 in analyses, 43%−50% Age: M = 23 (SD = 13.1) Recovered, M = 28 (SD = 15.2) Not recovered. Range 10–71 Setting: outpatient concussion/ neurosurgery clinic	Retrospective cohort study, with questionnaire follow-up patients needed to have post-concussion syndrome at 3 months to be included in this study. It compared those with pcs who eventually recovered to those with pcs who did not recover. Outcome: time to recovery (either in chart or self-reported) Findings: there was no difference in the portion of patients with pre-injury migraines in the recovered vs. not recovered groups (*p* = 0.94). Effect size = NR	No	Years
Howell (2016) 27230721	*N* = 1,050, 318 in analysis, 21% ages 8–12; 38% ages 13–18 Age: M = 14.7 (IQR = 12.9–16.2), Range = 8–18 Setting: sports concussion clinic	Retrospective cohort study Outcome: persistent symptoms >28 days vs. symptom resolution <28 days Findings: ages 8–12: 24% of prolonged symptom group had migraine history vs. 4% of other group (*p* = 0.025). ages 13–18: 8% of prolonged symptom group had migraine history vs. 10% shorter recovery group (*p* = 0.81). Effect size = NR	Yes and No	28 days
Howell (2018) 30265817	*N* = 230, 230, 50% Age: M = 14.8, SD = 2.5, <19 years old. Setting: sports concussion clinic	Retrospective cohort study Outcome: symptom resolution >28 days vs. symptom resolution ≤ 28 days; time to symptom resolution	No	28 days
		Findings: 19% of those with pre-injury migraines had a prolonged recovery, compared to 9% of those without a history of migraines. This was not significant in univariable analyses (*p* = 0.09) or multivariable analyses (*p* = 0.48; OR = 1.88; 95% CI = 0.73–4.80).		
Howell (2019) 30994475	*N* = 351, 351, 33% Age: M = 14.9, SD = 2.2, IQR = 13.1–16.1 Setting: sports concussion clinic	Prospective cohort study Outcome: time to symptom resolution. Findings: those with a history of headaches or migraines did not have a longer symptom resolution time in a univariate analysis, *p* = 0.76, HR = 1.05 (95% CI = 0.76–1.46).	No	Until recovery (Md = 23 days, IQR = 16–32)
Kontos (2019) 31479086	*N* = 314, 314, 36% Age: Range: 12–23 Setting: sports concussion clinic	Prospective cohort study Outcome: time to return to play Findings: those with pre-injury migraines had a longer recovery time compared to those without pre-injury migraines (HR = 0.68, 95% CI = 0.48–0.94). The mean recovery time was 5.5 weeks (SD = 4.2) for the pre-injury migraine group and 4.0 weeks (SD = 2.7) in those without migraines. Pre-injury migraine history was also associated with recovery in a multivariable model (HR = 0.69, 95% CI = 0.49–0.99)	Yes	Until recovery (M = 27.5 days, SD = 25 days)
Kontos (2020) 31904763	*N* = 162, 162, 56% Age: M = 15.3, SD = 1.6, range = 12–22 Setting: sports concussion clinic	Retrospective cohort study Outcome: time to return to play >30 days Findings: A history of pre-injury migraines was not associated with a prolonged recovery (statistics not provided). Effect size = NR.	No	Until recovery (M = 57 days, SD = 56, range = 9–299)
Lau (2011) 21285444	*N* = 108, 108, 0% Age: M = 16.12, 15.9, SD = 1.2, 1.2, Range = NR Setting: high school	Prospective cohort study Outcome: return to play; >14 days Findings: those with and without pre-injury migraines did not have differences in recovery time (p=0.557). Effect size = NR.	No	14 days (RTP variable)
Lau (2011) 21712482	*N* = 107, 107, 0% Age: M = 16.02, SD = 1.22, Range = 13–19 Setting: high school	Prospective cohort study Outcome: protracted recovery (≥21 days) vs. short recovery ( ≤ 7 days) Findings: there was no statistically significant difference the protracted (8.3%) and rapid recovery groups (4.8%) on preinjury history of migraine (*p* = 0.66). Effect size = NR	No	Until recovery (mean = 13 days, SD = 9 days)
Lau (2012) 21841522	*N* = 108, 108, 0% Age: M = 16.12; 15.9; 15.94, SD = 1.2, Range = NR Setting: high school	Prospective cohort study Outcome: cognition; short recovery ( ≤ 14 days) vs. protracted recovery (>14 days) Findings: there was not a statistical difference between the short and protracted recovery groups based on pre-injury history of migraines (*p* = 0.94). Effect size = NR	No	14 days cutoff (RTP variable)
McDevitt (2015) 26502998	*N*=87, 87, 26% Age: M = 19.47, SD = 6.02, Range = NR Setting: hospital concussion program	Case series study (i.e., prospective cohort) Outcome: return to play, prolonged recovery (>60 days) Findings: those with migraines did not have a higher likelihood of prolonged recovery (*p* = 0.825). Effect size = NR	No	Many months
Meehan (2013) 23628374	*N* = 182, 182, 36% Age: M = 15.2, SD = 3.04, Range = 7.6–26.7 Setting: clinic	Prospective cohort study Outcome: duration of symptoms. prolonged recovery (>28 days vs. <28 days) Findings: those with migraines did not have a higher likelihood of prolonged recovery (*p* = 0.98). Effect size = NR	No	28 days
Meehan (2014) 25381296	*N* = 531, 531, 38% Age: M = 14.6, SD = 2.9, Range = 7–26 Setting: clinic	Prospective cohort Outcome: clinical recovery (symptoms, cognition, balance); prolonged recovery (>28 days vs. <28 days) Findings: those with migraines did not have a higher likelihood of prolonged recovery (*p* = 0.259). Effect size = NR	No	28 days
Meehan (2016) 26718812	*N* = 131, 64 in analysis, 47% Age: M = 21, SD = 2, Range = 18–27 Setting: concussion clinic	Prospective cohort Outcome: symptom duration; prolonged recovery (>28 days vs. <28 days) Findings: of the 4 people who reported pre-injury migraines, 2 got better within 28 days and 2 did not get better within 28 days (*p* = 0.62). Effect size = NR	No	28 days
Miller (2016) 26684762	*N* = 294, 294, 23% Age: M = 13.7; 12.6, SD = 2.5; 2.5, Range = 6–18; 4–18 Setting: clinic	Case-control study (2 subgroups analyzed separately) Outcome: time to symptom resolution, prolonged recovery (>28 days) Findings: With SCAT2: OR = 0.71 (0.14–3.65), *p* = 0.68l Without SCAT2: OR = 7.77 (0.88–68.56), *p* = 0.07; history of migraines were not associated with symptoms lasting longer than 28 days.	No	28 days
Morgan (2015) 25745949	*N* = 120, 120, 51% Age: M = PCS 14.9; control 14.8, SD = 2.1; 2.0, Range = NR Setting: database, high school	Retrospective case-control study Outcome: development of PCS Findings: development of PCS was not predicted by a history of migraines (*p* = 0.088). 20% of the PCS group had a history of migraines, while 8.8% of the control patients had a history of migraines. Effect size = NR	No	PCS diagnosis made at 3 months
Neidecker (2021) 33512393	*N* = 182, 182, 39.5% Age: M = 15.3, SD = 1.4, Range = 11–18 Setting: single sports medicine practice	Retrospective cohort study (chart review) Outcome: symptom recovery Findings: those with pre-existing migraines/frequent headaches appeared to have a slightly higher likelihood of having symptoms at 5 days post injury (boys: *n* = 9/14; 64%) and 7 days post injury (girls: *n* = 10/18; 56%) than children without migraines/frequent headaches (boys: *n* = 51/96, 53%; girls: *n* = 20/54, 37%). This paper did not compare the groups. Based on the raw data from the article, we calculated chi-squared values and they were not significant (boys: χ^2^ = 1.29, *p* = 0.26; girls: χ^2^ = 1.90, *p* = 0.17). Effect size: NR.	No	5 days for boys and 7 days for girls
Nelson (2016) 27164666	*N* = 2,055, 127, 20% Age: NR Setting: high school and university	Prospective cohort study Outcome: symptom recovery Effect size: HR = 1.38 (0.67–2.84), *p* = 0.38	No	Approximately 1 week for most patients; follow-up continued to 45 days
Popovich (2021) 30768444	*N* = 126, 126, 29% Age: M = 15.3 (range: 8–19) Setting: university sport concussion center	Retrospective cohort study Outcome: time to return to play Findings: history of migraine was not associated with concussion recovery time, HR = 0.82, 95% CI = −0.49–1.35, *p* = 0.43	No	Return to play (several weeks)
Sinnott (2019) 31521485	*N* = 50, 50, 43% Age: M = 15.1, SD = 2.1, range: 12–20 Setting: sports concussion clinic	Prospective cohort study Outcome: vestibular symptoms change between acute post-injury visit and follow-up visit. Findings: having a pre-injury migraine history was associated with persistent vestibular impairments at the second visit (χ2 = 6.93, *p* = 0.03). Of the 8 patients who had pre-injury migraines, 3 did not have vestibular impairments at the first visit. Of the 5 with initial vestibular impairment, all 5 had persistent vestibular issues at the second visit.	Yes	11–21 days
Terry (2019) 29732944	*N* = 48,000, 1,265, 42% Age: high school M = 16.1, SD = 1.3, College M = 20.1, SD = 1.4; Range 14–25 Setting: high school and university athletics	Prospective cohort study Outcome: return to School without accommodations; return to play Findings: compared to athletes without migraine histories, a lower percentage of athletes with a history of migraine had returned to school after 7 days (57% vs. 68%, *p* = 0.02), 14 days (75% vs. 88%, *p* < 0.001), and 21 days post-injury (89% vs. 94%, *p* = 0.03). There were no differences at 28 days post injury (*p* = 0.27). There were no differences regarding return to play between the migraine and non-migraine groups (*ps* = 0.12–0.95) Effect size: NR	Yes And No	Return to school, Md = 5, IQR = 2–9, Range = 0–168 Return to play, Md = 14, IQR = 10–22, Range = 0–207
Zemek (2016) 26954410	*N* = 2,584, 2,584, 39% Age: Md = 12, IQR = 9.2–14.6, Range = 5–18 Setting: multiple pediatric emergency departments	Multicenter prospective cohort study Outcome: 3 or more new or worse symptoms using the patient-reported postconcussion symptom inventory (compared to their recall of pre-injury symptoms). Findings/Effect Size: univariate OR=1.9 (1.4–2.6) from derivation cohort. 42.6% of the migraine group had symptoms at 28 days post injury, compared to 28.1% of the non-migraine group. Multivariate OR = 1.73 (1.24–2.43), *p* < 0.001	Yes	28 days

*IQR, Interquartile Range; M, mean; Md, Median; NR, not reported; PCS, Post-Concussion Syndrome/Post-Concussion Symptoms; RTP, return to play; SD, standard deviation. The four Lau studies appeared to use the same sample and completed very similar analyses*.

**Table 2:** Methodological review related to pre-injury migraines and concussion outcome.

							**Family history**		
**First Author (Year), PMID**	**Definition/Assessment of migraine/Headache (self-report, parent report, physician, medical records)**	**Number with migraines**	**Migraine frequency**	**Migraines clearly differentiated**	**Age of onset**	**Medication status**	**Reported**	**Findings**	**Part of hypotheses**
Aggarwal (2017), 29058480	Personal and family migraine history; method unclear	9	NR	Yes	NR	NR	Combined	Note A	Yes
Beauchamp (2019), 31050335	Parent Report	39	NR	Yes	NR	NR	NR	NR	Somewhat
Eagle (2020), 32330895	Self-reported history of headache or migraine	82	NR	No	NR	NR	NR	–	Somewhat
Eisenberg (2013), 23753087	Personal & family migraine history: self-report with optional parental assistance (*via* a questionnaire). Post-concussion symptoms: RPSQ.	29	NR	Yes	NR	NR	Yes	Note B	No
Ellis (2015), 26359916	Medical records: retrospective chart review	16	NR	NR	NR	NR	NR	–	No
Fehr (2019), 29084034	Medical records: retrospective chart review	53	NR	NR	NR	NR	NR	–	No
Hiploylee (2017), 27784191	Self-report *via* questionnaire	NR	NR	Yes	NR	NR	NR	–	No
Howell (2016), 27230721	Self-report *via* questionnaire	Ages 8–12: 7 Ages 13–18: 23	NR	Yes	NR	NR	NR	–	Somewhat
Howell (2018), 30265817	Self-report of physician-diagnosed migraines *via* questionnaire	29	NR	Yes	NR	NR	NR	–	Yes
Howell (2019), 30994475	Headache or migraine *via* self-report *via* questionnaire	96	NR	No	NR	NR	NR	–	Somewhat
Kontos (2019), 31479086	Self-report of medical diagnosis of migraine	41	NR	Yes	NR	NR	NR	–	Somewhat
Kontos (2020), 31904763	Self-report during standardized clinical interview	57	NR	Yes	NR	NR	NR	–	No
Lau (2011), 21285444	Self-report *via* ImPACT	11	NR	Yes	NR	NR	NR	–	No
Lau (2011), 21712482	Self-report *via* ImPACT	11	NR	Yes	NR	NR	NR	–	No
Lau (2012), 21841522	Self-report *via* ImPACT	11	NR	Yes	NR	NR	NR	–	No
McDevitt (2015), 26502998	Self-report on a standardized initial evaluation	10	NR	NR	NR	NR	NR	–	No
Meehan (2013), 23628374	Self-report *via* standardized intake forms	19	NR	Yes	NR	NR	NR	–	No
Meehan (2014), 25381296	Self-report *via* intake forms	42	NR	Yes	NR	NR	NR	–	Somewhat
Meehan (2016), 26718812	Self-report intake forms	4	NR	Yes	NR	NR	NR	–	Somewhat
Miller (2016), 26684762	Retrospective self and family report	With SCAT 2: 8 Without SCAT 2: 6	NR	Yes	NR	NR	NR	–	No
Morgan (2015), 25745949	Electronic medical records and a health history form	15	NR	Yes	NR	NR	Yes	Note C	No
Neidecker (2021), 33512393	Electronic medical record review	57	NR	No; Note D	NR	NR	NR	–	No
Nelson (2016), 27164666	Baseline and postinjury clinical exams, which consisted of heath history	8, [6.5% of sample, *n* = 127]	NR	Yes	NR	NR	NR	–	No
Popovich (2021), 30768444	Electronic medical record review	25	NR	Yes	NR	NR	NR	–	No
Sinnott (2019), 31521485	Clinical interview	8	NR	Yes	NR	NR	NR	–	No
Terry (2019), 29732944	Self-Report to Athletic Trainer	117	NR	Yes	NR	NR	NR	–	Yes
Zemek (2016), 26954410	Physician diagnosed preinjury history of Migraine based on parent/child self-report	Derivation = 204 Validation = 119	NR	Yes	NR	NR	Yes	Note E	Somewhat

*Migraine Frequency refers to number of migraines in past month or year. Migraines clearly differentiated means that they are clearly differentiated from other headache disorders. Part of the hypotheses “Somewhat” means that it was combined with other variables. Note A: Family history and personal history of migraine were combined. Note B: Family history of migraine (n = 91) not significantly associated with outcome (p = 0.62). Note C: Development of PCS was associated with a family history of migraine (p = 0.003). 35% of the PCS group had a family history of migraines, while 11.3% of the control group had a family history of migraines. Note D: “Migraines or frequent headaches”. Note E: Family history of migraine Deviation n = 931 (Validation n = 505) was not a significant predictor of symptoms at 28 days (p = 0.08). ED, Emergency Department; ImPACT, Immediate Post-Concussion Assessment and Cognitive Testing; Md, Median; NR, Not Reported; OR, Odds Ratio; PCS, Post-Concussion Syndrome/Post-Concussion Symptoms; HR, Hazard Ratio; RPSQ, Rivermead Post-Concussion Symptom Questionnaire; SCAT, Sport Concussion Assessment Tool*.

### Level of Evidence and Risk for Bias Assessment

Level of evidence ratings and results of the QUIPS are summarized in Table 3. The level of evidence for most studies was rated as 3 (i.e., 22/25 studies), and three studies were rated a 4 making the average level of evidence 3.1. Regarding risk of bias, most studies (21/25; 84%) were rated as having a moderate overall risk of bias. Two studies (8%) had a low overall risk of bias and two studies (8%) had a high overall risk of bias. Seven studies (28%) were rated as having low risk of selection bias and 17 studies (68%) were at moderate risk of selection bias due to inadequate methods to identify the target population and unclear or inadequate participation by eligible individuals. Almost all studies (92%) were at moderate risk of prognostic factor misclassification predominantly due to an unclear definition and method of assessing the prognostic factor (i.e., pre-injury migraine). Nine studies (36%) were at low risk of bias due to outcome measurement and 15 studies (60%) were at moderate risk of bias due to outcome measurement that was attributable to uncertainty about the reliability of the outcome measurement method (e.g., participants had to recall the last date on which they experienced post-concussion symptoms). Almost all studies (21/25; 84%) were at moderate risk of bias regarding the potential presence of confounding variables because such variables were not incorporated in analyses involving pre-injury migraines and outcome (e.g., biological sex, concussion history). Almost half of the studies (10/25; 40%) had low risk of outcome and analytical reporting bias while most other studies (14/25; 56%) had a moderate risk of bias due to potential incomplete reporting of study outcome measures and statistical analyses.

**Table 3:** Risk of bias, as rated by the Quality in Prognostic Studies (QUIPS), and oxford centre for evidence-based medicine level of evidence.

**First author (year)**	**Study participation**	**Study attrition**	**Prognostic factor measurement**	**Outcome measurement**	**Confounding**	**Statistical analysis & reporting**	**Overall judgment**	**OCEBM LOE (1–5)**
Aggarwal (2017)	Low	Moderate	Moderate	Moderate	Moderate	Moderate	Moderate	3
Beauchamp (2019)	Low	Moderate	Moderate	Low	Low	Moderate	Moderate	3
Eagle (2020)	Moderate	Moderate	High	Moderate	Moderate	Moderate	Moderate	3
Eisenberg (2013)	Low	Moderate	Moderate	Moderate	High	Moderate	Moderate	3
Ellis (2015)	Moderate	Moderate	Moderate	Moderate	Moderate	Moderate	Moderate	3
Fehr (2019)	Moderate	Moderate	Moderate	Moderate	Moderate	Moderate	Moderate	3
Hiploylee (2017)	High	High	Moderate	High	Moderate	Moderate	High	3
Howell (2016)	Moderate	Moderate	Moderate	Low	Moderate	Low	Moderate	3
Howell (2018)	Moderate	High	Moderate	Moderate	Moderate	Low	Moderate	3
Howell (2019)	Moderate	Moderate	Moderate	Moderate	Moderate	Moderate	Moderate	3
Kontos (2019)	Moderate	Low	Moderate	Low	Low	Low	Low	3
Kontos (2020)	Moderate	High	Moderate	Moderate	Moderate	Moderate	Moderate	3
Lau (2011)	Moderate	Moderate	Moderate	Low	Moderate	Moderate	Moderate	3
Lau (2011)								3
Lau (2012)								3
McDevitt (2015)	Moderate	Moderate	Moderate	Moderate	Moderate	Moderate	Moderate	4
Meehan (2013)	Moderate	Moderate	Moderate	Low	Moderate	Low	Moderate	3
Meehan (2014)	Moderate	Moderate	Moderate	Low	Moderate	Low	Moderate	3
Meehan (2016)	Moderate	Low	Moderate	Low	Moderate	Low	Moderate	3
Miller (2016)	Moderate	High	Moderate	Low	Moderate	Moderate	Moderate	4
Morgan (2015)	Low	Moderate	Moderate	Moderate	Moderate	Low	Moderate	4
Neidecker (2021)	Moderate	High	High	Moderate	Moderate	High	High	3
Nelson (2016)	Low	Low	Moderate	Moderate	Moderate	Low	Moderate	3
Popovich (2021)	Moderate	Moderate	Moderate	Moderate	Low	Moderate	Moderate	3
Sinnott (2019)	Moderate	Moderate	Moderate	Moderate	Moderate	Moderate	Moderate	3
Terry (2018)	Low	Moderate	Moderate	Moderate	Moderate	Low	Moderate	3
Zemek (2016)	Low	Low	Moderate	Low	Moderate	Low	Low	3

*LOE = Level of Evidence; OCEBM = Oxford Centre for Evidence-Based Medicine*.

### Pre-injury Migraines and Clinical Outcome: Overall Findings

Of the 25 studies, most (*k* = 19, 76%) did not find that pre-injury migraines were a risk factor for a worse clinical outcome following a sport-related concussion, two studies (8%) had both negative and positive findings, and four studies (16%) had positive findings; of note, one study did not conduct statistical analyses, (33) but based on the raw data reported in the paper, we conducted chi-squared tests and the *p*-values were >0.05; this study was included as one of the 19 studies with null results. Across all studies, 24% (*k* = 6/25) reported at least one result (e.g., symptom duration, time to return to play) showing an association between pre-injury migraine history and worse clinical outcome. There was a large amount of variability in how each study defined the outcome.

Likelihood ratios favored the null hypothesis with strong magnitude when you assume ideal study parameters and weak magnitude when you assume more realistic study parameters (see Table 4). When analyses were restricted to studies that included 20 or more and 50 or more individuals with a pre-injury history of migraine, 35.7% (*k* = 5/14) and 42.3% of studies (*k* = 3/7) had at least one positive finding, respectively. For studies that included over 50 people with pre-injury migraines, the likelihood ratios supported the alternative hypothesis (i.e., that there was a positive association between pre-injury migraine history and worse clinical outcome) with weak-to-moderate magnitude.

**Table 4:** Likelihood ratio estimates.

			**Likelihood ratio & hypothesis favored**	
	**Total Studies**	**Number of “Positive” Studies**	**80% Power, 5% Alpha**	**45% Power, 25% Alpha**
All Studies	25	6	429,001, null favored	1.66, null favored
Studies with Pre-Injury Migraine *N* ≥ 20	14	5	1.17, null favored	1.16, alternative favored
Studies with Pre-Injury Migraine *N* ≥ 50	7	3	8.05, alternative favored	1.69, alternative favored

“*Positive” refers to at least one statistically significant result (i.e., p ≤ 0.05). ^+^Higher likelihood ratios (LR) indicate increased likelihood in favor of the alternative hypothesis (i.e., pre-injury migraine is associated with clinical outcome); magnitude of LRs were characterized as follows: LR = 1 = no effect, LR between 1 and 8 = weak, LR > 8 and <32 = moderate, LR > 32 = strong; N, number of study participants in included studies*.

### Narrative Review of Studies

In prospective cohort studies examining high school athletes, similar rates of athletes with and without pre-injury migraines had returned to play at 14 days (30) and at 28 days post injury (19, 20). Similarly, a prospective cohort study that examined symptom recovery time in high school and college athletes did not find an association between pre-injury history of migraines and time to recover (21). In specialty sport concussion clinic studies, pre-injury migraines were not associated with a longer recovery [i.e., >28 days (14, 34, 35); self-reported symptom recovery (36–39)] with the median recovery time for the entire sample being between 23 and 40 days across three studies (37–39). Pre-injury migraines were, however, associated with prolonged vestibular-ocular difficulties at 11–21 days following concussion (40). Another study in a specialty sport concussion clinic showed that amongst younger children (i.e., ages 8–12), those with migraines had a higher likelihood of having symptoms at 28 days following injury (24%) compared to those without migraines (4%; *p* = 0.025); however, this finding was not apparent in youth ages 13–18 (8% vs. 10% prolonged recovery, *p* = 0.81) (41). A different adolescent concussion clinic study reported that those with a “history of headaches or migraines” (36% of the entire sample) showed a higher likelihood of prolonged recovery at 30 days (OR = 4.02) (42). Adolescents with concussion seen in university settings did not have a higher likelihood of prolonged recovery (43) or greater time to return to play (44). In a study that examined adolescent and young adult athletes recruited from a hospital-based concussion program, those with a pre-injury history of migraines were not more likely to have a prolonged recovery (i.e., return to play > 60 days; *n* = 2/10, 20%) compared to those who did not have a pre-injury history of migraines (*n* = 18/77, 23.4%; *p* = 0.83) (18).

In a prospective surveillance study that followed players in high school and collegiate athletics, those with migraines took a median of 6 days to return to academics without accommodations and 15.5 days to return to athletics fully, compared to 5 days (for academics) and 14 days (for athletics) in those without migraines (45). Those with and without pre-injury migraines did not differ on their time to return to academics or athletics (45) when recovery time was treated as a continuous variable. However, slightly lower percentages of athletes with migraine histories returned to academics after 7 days (57% vs. 68%, *p* =0.02), 14 days (75% vs. 88%, *p* < 0.001), and 21 days post injury (89% vs. 94%, *p* = 0.03) (45). When the authors stratified this analysis by gender, this effect was significant in girls and women with pre-injury migraines, but not boys and men (45).

In a retrospective chart review that examined whether high school athletes experienced prolonged recovery [post-concussion syndrome (PCS) diagnosis] at 3 months post injury, the development of PCS was not predicted by a history of migraines (*p* = 0.09) (13). However, pre-injury migraine was overrepresented in the PCS group, as one-fifth (20%) of the PCS group had a history of migraines, while 8.8% of the non-PCS patients had a history of migraines (13).

In one study of adolescents and young adults recruited from an emergency room setting (64% sports injuries), the median recovery time for those with a pre-injury history of migraines was 13 days, which matched the median recovery time of the entire sample (16). Additionally, a similar proportion of those with migraines had symptoms at 28 days post injury (35%) compared to the entire sample (32%; *p* = 0.72) (16). A different prospective study that examined sports concussions in patients ages 12–17 who presented to an emergency department did not show a higher likelihood of prolonged recovery (46). In contrast, another study examined youth ages 5–18 who presented to emergency departments across Canada and found a higher portion of the migraine group (42.6%) had symptoms at 28 days post injury, compared to 28.1% of the group without migraines (10). The association between pre-injury migraine and persistent symptoms was significant in both univariate and multivariate models.

Those with pre-injury migraines did not have a higher likelihood of developing a psychiatric diagnosis following a concussion compared to those without pre-injury migraines (17). One study examined patients who all had persistent symptoms for at least 3 months following their injury. Patients were sent a questionnaire regarding their recovery by mail months to years following their injury. Some patients reported they never recovered. There was no difference in the proportion of patients with pre-injury migraines in the recovered vs. the not recovered groups (*p* = 0.94) (32).

### Family History of Migraines and Clinical Outcome

Three studies assessed the association between a family history of migraines and recovery from concussion. One study examining high school athletes showed that the development of PCS at 3-months following injury was associated with a family history of migraine (*p* = 0.003), and that 35% of the PCS group had a family history of migraines compared to 11.3% of the control group (though, as noted above, in this same study the youth's own pre-injury migraine status *was not* associated with PCS) (13). The other two studies, both set in emergency departments, did not find an association between family history of migraines and time to recovery (*p* = 0.62) (16) or post-concussion symptoms at 28 days post injury (*p* = 0.08) (10). One study assessed if patients had a “personal or family history of migraine,” and did not find that migraines were predictive of time to symptom resolution (43).

### Sample Size

One study did not report the number of participants with migraines (32) The number of participants with migraines in each study ranged from 4 to 204, with an average of 42.3 participants with migraines (total = 1,015; median = 29; based on the 24 studies reporting their sample size). Ten of the 24 studies (41.7%) included fewer than 20 participants who had pre-injury migraines. The two studies with the largest sample sizes of people with pre-injury migraines [*n* = 117 (45) and *n* = 204 (10)] were two of the six studies that showed positive findings between pre-injury migraines and worse clinical outcome following concussion.

### Limited Clinical Information Relating to Pre-injury Migraines

All studies lacked basic clinical information related to pre-injury migraines, limiting the ability to understand how pre-injury migraines may be related to a worse outcome from a sport-related concussion. In 17 studies (68%), pre-injury history of migraines was assessed based on self- or parent-report, likely with a single item. It was not specified whether a definition of a migraine was provided to these athletes when they completed this question. Additionally, the specific wording related to this question was not clearly specified for all studies and thus likely differed across studies (e.g., “Has a physician treated you for migraines?,” “Do you have migraines?,” “Have you been diagnosed with a migraine disorder?”). Five studies (20%) used retrospective chart reviews, which may indicate that a diagnosis of migraine was rendered by a physician, but this cannot be determined from the information provided in the studies. Two studies (8%) based pre-injury migraine status on a clinical examination [one null (21), one significant (40)], and only three studies (12%) specified the migraines needed to be physician-diagnosed [two null (35, 36), one significant (10)]. No studies reported the age of onset for the migraines, current frequency/severity, or other important clinical features.

## Discussion

This systematic review identified 27 studies that examined the association between pre-injury migraines and outcome following concussion, of which 25 comprised unique samples. Across these studies, an average of 11.8% of athletes reported a pre-injury history of migraine. It is essential to appreciate that *only one study* was designed specifically to examine migraine as a risk factor (45). All other studies included migraine as a secondary or exploratory variable; some studies discussed their rationale for examining pre-injury migraines (10, 20, 34–37, 41–43, 46), while others did not (13, 14, 16–19, 21, 30, 32, 33, 38–40, 44). Most studies (*n* = 19/25, 76%) did not find a statistically significant association between having pre-injury migraines and a prolonged recovery or worse clinical outcome following concussion, two studies had both significant and null findings, and four studies showed only significant findings.

A few studies found an association between pre-injury migraine and clinical outcome from concussion. The largest study examined in this systematic review, conducted in emergency departments across Canada, was also one of the two studies with an overall risk of bias judgment of “low” using the QUIPS tool (10). In that study, 42.6% of youth with a history of pre-injury physician-diagnosed migraines had persistent symptoms 28 days following their concussion, compared to 28.1% of youth without pre-injury migraines (OR = 1.9, *p* < 0.001) (10). Moreover, in a multivariable model that also included demographic factors (e.g., age, sex), history of prior concussions with symptoms that lasted more than 1 week, and acute post-injury symptoms (e.g., poor balance; headache, noise sensitivity, fatigue, answering questions slowly), pre-injury migraine history was independently associated with the presence of symptoms 4 weeks following injury (OR = 1.7, *p* < 0.001) (10). In the only other study with an overall “low” risk of bias, which examined athletes ages 12–23, the authors reported that patients with a pre-injury history of migraines (*n* = 41) had a longer recovery time (M = 5.5 weeks, SD = 4.2), taking, on average, about a week and a half longer to recover compared to those without pre-injury migraines (M = 4.0 weeks, SD = 2.7) (37). This finding was present in a univariable analysis as well as a multivariable model that also included initial symptom burden, posttraumatic migraine symptoms, biological sex, and age (i.e., dichotomized as <8 years old) (43). The two other studies that found pre-injury migraines as significantly associated with outcome had important methodological limitations, like combining headache and migraine history (36% of the sample) (42), which limits the generalizability of their findings, or examining a very small sample of people with pre-injury migraines (i.e., *n* = 8 total, with *n* = 5 in the statistical analysis) using a non-traditional definition of recovery (i.e., vestibular deficits at 11–21 days post-injury) (40).

Two studies had mixed findings. An observational study of high school and collegiate athletes examined full return to academics without accommodations and athletics (45) This study had the second largest migraine sample and was the only study with a primary aim to examine pre-injury migraines as a risk factor for prolonged recovery; most other studies included a variety of other potential prognostic factors and analyzed pre-injury migraine status in an exploratory or secondary manner. In this study (45), there were no statistically significant differences in the overall recovery times between athletes with and without pre-injury migraines. However, a lower proportion of those with pre-injury migraines fully returned to school at 7, 14, and 21 days post injury (45). This finding extends a prior study that showed high school athletes with pre-injury migraines appear to have greater symptoms in the first 3 days following concussion compared to non-migraineurs (12). Taken together, these studies suggest that athletes with pre-injury migraines have greater acute symptomatology following concussion, which may influence proximal outcomes (e.g., return to school) more than distal outcomes (e.g., return to play). Previous studies have consistently shown that greater acute symptom burden (45) and the presence of acute headaches/migraines (45, 47) are associated with a prolonged recovery. The final study with positive findings (41) found that 71% (*n* = 5/7) of youth athletes with pre-injury migraines (ages 8–12) had symptoms >28 days compared to only 26% of youth athletes without migraines (*n* = 16/61), though this analysis was based a very small sample of migraineurs and the finding did not replicate in the age 13–18 group (41).

The majority of studies did not report statistically significant differences in concussion outcome based on pre-injury migraine status, regardless of how it was defined. However, one of the most notable things about this body of literature is the variability in methodological rigor and sample size. Many studies included fewer than 20 patients with pre-injury migraines (13, 14, 17, 18, 21, 30, 34, 40, 43) [in some analyses (41)], and several had <*10* patients with a migraine condition (21, 34, 40, 43) [in some analyses (14, 41)]. These studies likely lacked the statistical power to detect differences between groups and are unlikely to embody a representative or generalizable subsample of individuals with pre-injury migraine. Further, these studies had variable methods, such that there were a variety of outcomes examined (e.g., symptom resolution, return to play, return to academics, PCS diagnosis, development of a psychiatric disorder) at a variety of different time points. Empirical ratings of risk of bias in these studies were mostly classified as having a “moderate” risk of bias and only one study was classified as having a “low” risk of bias. Therefore, it is reasonable to interpret these null findings with caution, and that these methodological issues limit our current ability to render a conclusion about the relationship between pre-injury migraines and concussion outcome with certainty.

### Potential Mechanisms

The mechanism by which some athletes with pre-injury migraines might have a longer or more complicated recovery from a concussion is not fully understood, but there are several noteworthy findings that suggest pre-injury migraines may be a risk modifier for a complicated concussion recovery. In both athletes and civilians, those with pre-injury migraines are more likely to have headaches and migraines following a concussion (48, 49) and post-injury headache and migraine are independent risk factors for a prolonged recovery from concussion (10, 50–54). Additionally, primary migraine disorders and post-traumatic headaches (which often manifest as having migraine-features) have similar pathophysiological profiles, involving alterations in ion flow, cerebral metabolism, and neurotransmitters/neuropeptides (55–59). It is possible that some of the pathophysiological processes underlying primary migraine disorders are more vulnerable to trauma, leading to an amplified neurovascular response (60). This could lead to worse acute symptoms following concussion for those with pre-injury migraines (12), and worse acute symptoms is one of the most consistent and robust predictors of prolonged concussion recovery (1).

### Limitations

There are several important limitations to consider when interpreting the results of this systematic review. In most studies, pre-injury migraine status was based on a single self-report item; very few studies used data based on a clinical examination by a trained medical professional or a structured clinical interview for headaches [e.g., the Headache-Attributed Restriction, Disability, Social Handicap and Impaired Participation (HARDSHIP) questionnaire and its diagnostic algorithm (61)]. Other clinical factors, such as the age of onset of the migraine disorder or pre-injury migraine frequency, were not reported in any of these studies. Thus, those athletes with pre-existing migraines are likely to be heterogeneous in their migraine frequency and medication status, which may affect the study outcome. It is also important to consider that there might be a gender-by-migraine interaction girls/women are more likely to have migraine conditions (62), report more symptoms during baseline preseason testing (9), have a higher prevalence of headaches/migraines following concussion (63), and might be at risk for prolonged recovery compared to boys/men (1). Thus, stratifying analyses in future migraine studies by gender may be useful. Further, those with pre-injury migraines, overall, report a greater severity of concussion-like symptoms and higher symptom provocation scores on vestibular/ocular-motor screens in the absence of a recent concussion during pre-season baseline testing (9, 11). It is possible that when athletes with pre-injury migraines sustain a concussion, some of their prolonged post-injury symptoms and functional difficulties may be misattributed solely to the effects of concussion, but may actually reflect a variety of factors associated with having a migraine disorder more broadly. As noted earlier, many studies analyzed a small number of people with pre-injury migraines and were likely underpowered to detect statistically significant differences between groups. Additionally, due to the variability across studies and their outcomes, quantitative analyses or meta-analytic synthesis of effect sizes could not be conducted. Lastly, like most systematic reviews, only published studies were examined. There is a chance that these conclusions are subject to publication bias.

### Knowledge Gaps and Directions for Future Research

There are several knowledge gaps that could be addressed in future research studies that examine the association between pre-injury migraines and clinical outcome from concussion. Many studies in this review recruited participants from specialty concussion clinics or hospital settings, while the most common setting in the United States for youth to receive a concussion diagnosis is from a primary care provider/pediatrician (64). Studies that sample individuals from specialty concussion clinics or emergency departments may represent a select and non-representative subset of individuals who sustain concussion, such that those who receive hospital care or specialty concussion services may have had more severe injury characteristics and/or are experiencing an atypical recovery. Designing studies that sample patients from more naturalistic and representative settings, such as primary care offices or prospectively following athletes *via* an injury surveillance system, will increase generalizability.

Further, studies should ensure they sample enough participants with migraines to achieve the statistical power necessary to detect differences between groups, if present. Additionally, future studies could more comprehensively describe the clinical characteristics of those with pre-injury migraines, by including empirically-supported migraine assessments, as well as measuring and reporting pre-injury migraine frequency, current migraine medication status, age of migraine onset, and pre-injury migraine functional impairment and disability. Future studies may also wish to examine the interplay between pre-injury migraines, prior concussions, and concussion recovery. It is possible that an athlete might develop a chronic migraine condition following a prior concussion, return to play, and then sustain a subsequent concussion. It will be important to study the clinical outcomes and recovery from concussion among these individuals.

The extant literature to date is inconclusive as to whether pre-injury migraine clearly confers risk for worse clinical outcome following a sport-related concussion—but it seems very likely that, at minimum, a subgroup of student athletes with pre-injury migraine are at increased risk. This would be most obvious for those who develop a mixed phenotype of post-traumatic headache, including worsening of their migraine condition, combined with difficulties with post-injury anxiety or depression. In the future, better prognostication—based on multiple possible risk factors such as *pre-injury* migraine, pre-injury mental health problems, and *acute* post-traumatic headache severity and the severity of other post-concussion symptoms—might, if implemented during the first few days following injury, allow for early targeted precision rehabilitation aimed at promoting a swifter and durable clinical recovery.

## Data Availability Statement

The original contributions presented in the study are included in the article, further inquiries can be directed to the corresponding author.

## Author Contributions

DT and GI conceptualized the study. DT, GI, AG, and NC screened abstracts. DT, FB, NH, AG, and NC extracted data and/or rated included studies. DT, FB, NH, NC, and GI all wrote portions of the manuscript. All authors contributed to the article and approved the submitted version.

## Funding

This study was funded in part by the National Football League for a program of research entitled The Spectrum of Concussion: Predictors of Clinical Recovery, Treatment and Rehabilitation, and Possible Long-Term Effects (PI Iverson). The funder was not involved in the study design, collection, analysis, interpretation of data, the writing of this article or the decision to submit it for publication. GI acknowledges unrestricted philanthropic support from the Mooney-Reed Charitable Foundation, Heinz Family Foundation, Boston Bolts, National Rugby League, ImPACT Applications, Inc., and the Spaulding Research Institute. None of the above entities were involved in the study design, collection, analysis, interpretation of data, the writing of this article, or the decision to submit it for publication.

## Conflict of Interest

GI serves as a scientific advisor for NanoDx® Inc., Sway Operations, LLC, and Highmark, Inc. He has a clinical and consulting practice in forensic neuropsychology, including expert testimony, involving individuals who have sustained mild TBIs (including athletes). He has received research funding from several test publishing companies, including ImPACT Applications, Inc., CNS Vital Signs, and Psychological Assessment Resources (PAR, Inc.). He has received research funding as a principal investigator from the National Football League, and salary support as a collaborator from the Harvard Integrated Program to Protect and Improve the Health of National Football League Players Association Members. DT has served as a consultant for REACT Neuro, Inc., and has a consulting practice in forensic neuropsychology involving individuals who have sustained mild TBIs (including athletes). AG serves as a scientific advisor for HitIQ, Ltd. He has a clinical practice in neuropsychology involving individuals who have sustained sport-related concussion (including current and former athletes). He is a contracted concussion consultant to Rugby Australia. He is a member of the World Rugby Concussion working group and the Australian Football League (AFL) Concussion Scientific Advisory Committee. He has received travel funding or been reimbursed by professional sporting bodies, and commercial organisations for discussing or presenting sport-related concussion research at meetings, scientific conferences, workshops, and symposiums. He has received research funding from the National Rugby League (NRL) and the Australian Institute of Sport (AIS) for the Retired Professional Rugby League Players Brain Health research program. Other previous research funding support includes: the NSW Sporting Injuries Committee, the Brain Foundation (Australia), and the Australian-American Fulbright Commission (Postdoctoral Award). He has received salary support from the Hunter Medical Research Institute (HMRI). The remaining authors declare that the research was conducted in the absence of any commercial or financial relationships that could be construed as a potential conflict of interest.

## Publisher's Note

All claims expressed in this article are solely those of the authors and do not necessarily represent those of their affiliated organizations, or those of the publisher, the editors and the reviewers. Any product that may be evaluated in this article, or claim that may be made by its manufacturer, is not guaranteed or endorsed by the publisher.
